# Proteomics-based screening of the target proteins associated with antidepressant-like effect and mechanism of nimesulide

**DOI:** 10.1038/s41598-020-66420-z

**Published:** 2020-07-06

**Authors:** Wen Luo, Ying Luo, Junqing Yang

**Affiliations:** College of Pharmacy, Chongqing Medical University, Chongqing Key Laboratory of Biochemistry and Molecular Pharmacology, Chongqing, 400016 China

**Keywords:** Pharmacology, Neuroscience, Proteomics

## Abstract

Nimesulide is an inhibitor of COX-2 with antioxidant and anti-inflammatory effects. However, few studies have explored the antidepressant mechanism of nimesulide. Here, we evaluated the therapeutic effects of nimesulide on CUMS rats. iTRAQ technology was used to identify the differentially expressed protein in the hippocampus between CUMS and nimesulide-treated rats to identify the possible molecular mechanism of its effects. We found that nimesulide had positive effects on depressive-like behaviors and inflammatory factors in depressed rats. Using proteomics technologies, we screened 16 differentially expressed proteins in CUMS-exposed rats after nimesulide treatment, 5 of which were related to inflammation. Overall, these results show that nimesulide might mediate its antidepressant effect on depressed rats through the inhibition of oxidative stress inflammatory response.

## Introduction

Depression is reported to be associated with many health problems like low mood, cognitive impairment, and even suicidal behaviours^1^. With the increase in competitive pressure in modern society, the number of people suffering from depression is growing day by day^2^. Furthermore, depression may induce substance abuse^3,4^ and suicide^5^. Brain imaging studies showed a reduced hippocampus volume of depressed patients^6^. However, a disadvantage of conventional antidepressants is a slow onset of antidepressant action. Therefore, the pathogenesis of depression needs further studies to search for new therapeutic targets.

Substantive evidence shows inflammation being a driver of neuropsychiatric symptoms by negatively impacting on neuronal proliferation, survival, and differentian^7–9^. A population-based, prospective cohort study in Denmark followed a total of 3.56 million people for 24 years and found that those who were hospitalized for an infection or visited a hospital for treatment of an autoimmune disease had a significantly higher risk for developing a depressive disorder^10^. The cyclooxygenase (COX) −2 catalyzes the synthesis of prostaglandins (PGs) and induces inflammation responses^11^. The COX pathway potentiates the inflammatory process and may exacerbate brain inflammation and injury. Clinical studies found that enhanced COX-2 expression in patients with neuropsychiatric diseases such as depression and Alzheimer’s disease^12–14^. Consistent with these results, an animal model of depression in rats demonstrated that the upregulation of COX-2 may induce hyperactivity in inflammatory responses and lead to depression-like behavior^15^. Interestingly, the COX-2 inhibitor can relieve depressive-like behaviors by inhibiting inflammatory factors^16^. In people with major depression, a randomized, double-blind study found compared reboxetine plus celecoxib with reboxetine plus placebo a significant therapeutic effect of celecoxib^17^. These results point out that a strong correlation between COX-2 and depression.

Fluoxetine is the first selective serotonin reuptake inhibitor antidepressant with the longest half-life and high bioavailability. Moreover, fluoxetine is one of the most widely prescribed antidepressants in clinical settings and has been experimentally widely used for studying depression^18^. As one of only two antidepressants, fluoxetine was approved for depression in youth by the United States Food and Drug Administration (FDA)^19^. Besides, fluoxetine was used as the control in a large number of animal experiments^20,21^.

Isobaric tags for relative and absolute quantification (iTRAQ) is an advanced technique used for identifying biomarkers and examining pathophysiological processes of various diseases^22^. In our study, we used iTRAQ to screen target proteins regulated by nimesulide in the hippocampus of CUMS rats, to provide insight into the pathogenesis of depression and the role of nimesulide in depression.

## Materials and methods

### Animals

Male Sprague-Dawley rats (180–220 g) were purchased from the Laboratory Animal Center of Chongqing Medical University. They were housed in groups of 5 each cage at constant room temperature (25 ± 2 °C) with a 12 h light and dark cycle. Food and water were continually available for 1 week as rats adapted to the environment. All procedures were performed following Chongqing Science and Technology Commission guidelines for animal research and approved by the Chongqing Medical University Animal Care Committee.

### Drugs and treatment groups

A total of 80 rats were randomly divided into a control group (CON, n = 20) and a CUMS-exposed group (n = 60). CUMS-exposed rats (n = 60) were further subdivided into the following 3 groups, with 20 rats each group: CUMS model group (CUMS), fluoxetine+ CUMS group (FLX), and nimesulide+ CUMS group (NIM). The dose of nimesulide for rats was derived from a human dose by following a conversion equation^23^. The rats in the NIM and FLX groups were treated with nimesulide (Hubei Jianyuan Chemical Co., Ltd. China) and fluoxetine (Wuhan Sheng Tianyu Biotechnology Co., Ltd. China) through oral gavage at a dose of 12 mg/kg/d and 3 mg/kg/d, respectively for 21 days. The rats in the CON and CUMS groups received an equal volume of 0.5% sodium carboxymethylcellulose (CMC-Na) (National Chemical Reagent, China) through oral gavage for 21 days. Behavioral tests were performed following drug intervention.

### CUMS procedure

The CUMS procedure was conducted according to the previous study^24^. All of these stressors were randomly arranged, including tail pinching for 1 min, 4 °C cold water swimming for 5 min, 45 °C hot water swimming for 5 min, fasted (food and water) for 24 h, cage tilting at 45 ° for 24 h, wet bedding for 24 h, inverted day/night cycle and odor stimulation. Rats were individually exposed to the stressors in random order once a day for 21 days. No single stressor was performed consecutively.

### Open-field test (OFT)

The OFT was performed with minor modifications as described previously^25^. Rats were individually placed in the center of a black box (100 cm × 100 cm × 50 cm) in a dark and quiet room with a 5-m visibility distance. Horizontal (crossing times) and vertical (rearing times) exploratory activity were measured for a 5-min session. The apparatus was cleaned with detergent before each test session to remove any olfactory cue.

### Forced swimming test (FST)

The FST was conducted as previously described with minor modifications^26^. Rats were individually placed in a transparent cylindrical bucket (45 cm in height and 25 cm in diameter) filled with water (23 ± 2 °C) to a depth of 30 cm. Total immobility time in 6 min was calculated. The water was completely replaced after each test.

### Enzyme-linked immunosorbent assay (ELISA)

The rats’ hippocampus of each group was removed. The levels of interleukin-1β (IL-1β), interleukin-6 (IL-6) and tumor necrosis factor-α (TNF-α) in the hippocampus were quantified by ELISA following the instruction manuals (Haiyun Biological Biotechnology Co., Ltd).

## iTRAQ of CUMS hippocampus samples

### Protein extraction and iTRAQ sample labeling

The iTRAQ procedure was provided by the Institute of Life Sciences of Chongqing Medical University. Removed hippocampus tissues of 3 rats/group were immediately snapped-frozen within liquid nitrogen and stored in a refrigerator (−80 °C) until isobaric labeling. The 5 μL phosphatase inhibitor, 1 μL protease inhibitor, and 10 μL phenylmethylsulfonyl fluoride (PMSF) were added to lysis buffer, and the mixture was homogenized. The lysis buffer and glass homogenizer were then on ice. The cryopreserved rat hippocampus tissue was washed with phosphate Buffered Saline (PBS), and lysis buffer was added to rat hippocampal tissue, which was manually ground to a homogenate using a glass homogenizer. Next, we added trichloroacetic acid (TCA)-ice acetone to the homogenate and centrifuged it at 3000 rpm in 30 min at 4 °C after precipitation at 20 °C for 2 h, ultimately taking the precipitate without the supernatant. The precipitate was then added to acetone, as described previously, precipitating for 30 min at −20 °C and centrifuging at 3000 rpm for 30 min at 4 °C. Then, the supernatant was removed and the precipitate was kept. The above operation was repeated until the precipitate became white and stored at −80 °C.

### Strong cation exchange chromatography (SCX) fractionation and liquid chromatography-tandem mass spectrometry (LC-MS/MS) analysis

The extracted protein was subjected to reductive alkylation, which opening the disulfide bond to fully hydrolyze the protein. The protein concentration was then determinate by the Bradford method and detected by SDS-polyacrylamide gel electrophoresis (SDS-PAGE). Peptides were labeled with iTRAQ Reagent Kit according to the manufacturer’s protocol (Applied Biosystems, USA) after digesting samples into peptides using trypsin, and the labeled peptides were mixed in equal amounts. The mixed peptide was then pre-separated on SCX and subjected to LC-MS/MS analysis.

### Bioinformatics analysis

Proteins with a greater than 1.2- fold change or a fewer than 0.83- fold change and a P-value < 0.05 were considered differentially expressed. All significant proteins were uploaded to DAVID for Gene Ontology (GO) annotations of biological process, molecular function, and cellular component. Pathway enrichment analysis was performed based on the KEGG database.

### Statistical analysis

All the data were presented as mean ± standard error (SEM) and analyzed with GraphPad Prism 5.0. The data were analyzed in a One-way ANOVA followed by Dennett’s t-test and P < 0.05 was considered significant.

## Results

### Effects of nimesulide on depressive-like behavior in CUMS rats

**Figure 1:**
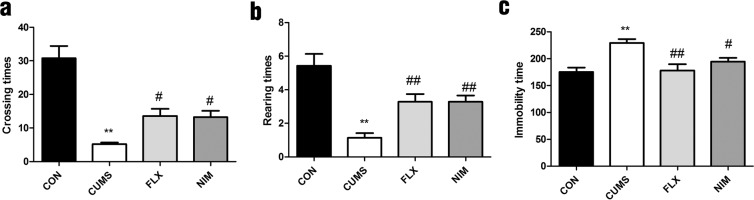
Effects of nimesulide on depressive-like behaviors in CUMS rats. (**a**) Nimesulide improved crossing times in CUMS rats. (**b**) Nimesulide improved rearing times in CUMS rats. (**c**) Nimesulide decreased immobility time in CUMS rats. All data are presented as mean ± SEM, n = 10 rats/group. ^**^p < 0.01 vs. CON; ^##^p < 0.01, ^#^p < 0.05 vs. CUMS. One-way ANOVA followed by Dennett’s t-test.

For the CUMS model, we used OFT and FST tests for evaluating depressive-like behaviors. As expected, we observed that CUMS induced significant behavioral changes (F_3,52_ = 21, p < 0.01; F_3,52_ = 13, p < 0.01; F_3,44_ = 8.3, p < 0.01; Fig. 1a–c). Compared with CUMS group, we found that nimesulide increased the horizontal (crossing times) and vertical (rearing times) movements (F_3,52_ = 21, p < 0.05; F_3,52_ = 13, p < 0.01; Fig. 1a–b). Moreover, we also found that nimesulide significantly shortened immobility time compared with CUMS group (F_3,44_ = 8.3, p < 0.05; Fig. 1c).

### Effects of nimesulide on the levels of the inflammatory factors in CUMS rats

**Figure 2:**
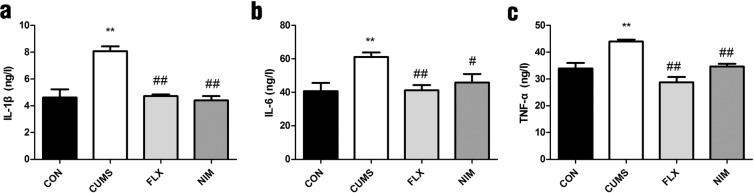
Effects of nimesulide on levels of cytokines in CUMS rats. (**a**) Nimesulide decreased the levels of IL-1β in CUMS rats. (**b**) Nimesulide decreased the levels of IL-6 in CUMS rats. (**c**) Nimesulide decreased the levels of TNF-α in CUMS rats. All data are presented as mean ± SEM, n = 3 rats/group. ^**^p < 0.01 vs. CON; ^##^p < 0.01, ^#^p < 0.05 vs. CUMS. One^*-*^way ANOVA followed by Dennett’s t-test.

Next, we evaluated the effect of chronic stress on inflammatory response. IL-1β, IL-6 and TNF-α levels were significantly increased after CUMS exposure (F_3,28_ = 21, p < 0.01; F_2,21_ = 10, p < 0.01; F_3,28_ = 16, p < 0.01; Fig. 2a–c). Nimesulide reversed the elevation of these inflammatory factors (F_3,28_ = 21, p < 0.01; F_3,28_ = 5.6, p < 0.05; F_3,28_ = 16, p < 0.01; Fig. 2a–c).

### Effects of nimesulide on hippocampal protein profiles in CUMS rats

**Figure 3:**
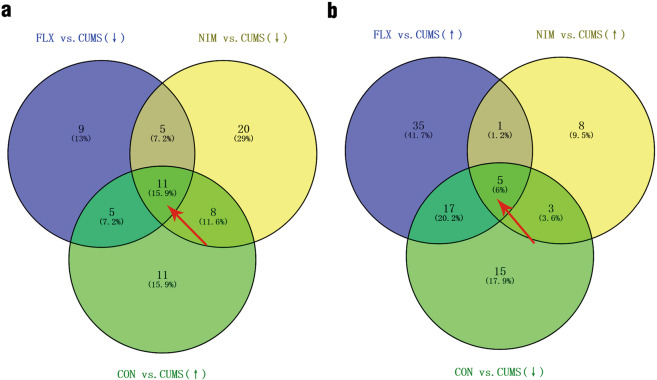
Venn diagram presents overlapping relationships and the numbers indicate differentially expressed protein counts. Overlapping regions of the Venn diagrams indicate differentially expressed proteins shared between or among corresponding groups. (**a**) The red short arrow indicates 11 overlappings differentially expressed proteins. Compared with the CUMS group, these proteins were downregulated by fluoxetine or nimesulide but upregulated in the CON group. (**b**) The red short arrow indicates 5 overlappings differentially expressed proteins. Compared with the CUMS group, these proteins were upregulated by fluoxetine or nimesulide but downregulated in the CON group.

Rats hippocampal samples labeled with iTRAQ reagents were analyzed by LC-MS/MS. A total of 153 proteins were found to be significantly changed, with 88 changed in groups FLX vs. CUMS, 61 changed in groups NIM vs. CUMS, 75 changed in groups CON vs. CUMS. The 16 differentially expressed proteins shared among groups FLX vs. CUMS, groups NIM vs. CUMS, and groups CON vs. CUMS, with 11 downregulated (Fig. 3a, Table 1) and 5 upregulated (Fig. 3b, Table 2). Of the 16 differentially expressed proteins, 5 were related to inflammation.

**Table 1:** Differentially expressed proteins were downregulated in the hippocampus of CUMS rats by nimesulide.

Accession	Protein description	Abbreviation	Fluoxetine/CUMS	Nimesulide/CUMS	CON/CUMS
ALDOC	Fructose-bisphosphatealdolase C	Aldoc	↓	↓	↑
KPCD	Protein kinase C delta type	Prkcd	↓	↓	↑
DHPR	Dihydropteridinereductase	Qdpr	↓	↓	↑
NFM	Neurofilament medium polypeptide	Nefm	↓	↓	↑
AATC	Aspartate aminotransferase, cytoplasmic	Got1	↓	↓	↑
NFL	Neurofilament light polypeptide	Nefl	↓	↓	↑
CALB2	Calretinin	Calb2	↓	↓	↑
SIR2	NAD-dependent protein deacetylase sirtuin-2	Sirt2	↓	↓	↑
MYO1D	Myosin-Id	Myo1d	↓	↓	↑
AMPL	Leucine aminopeptidase 3	Lap3	↓	↓	↑
S6A11	Sodium- and chloride-dependent GABA transporter 3	Slc6a11	↓	↓	↑

**Table 2:** Differentially expressed proteins were upregulated in the hippocampus of CUMS rats by nimesulide.

Accession	Protein description	Abbreviation	Fluoxetine/CUMS	Nimesulide/CUMS	CON/CUMS
AP2B1	AP-2 complex subunit beta 1	Ap2b1	↑	↑	↓
CANB1	Calcineurin subunit B type 1	Cnb1	↑	↑	↓
ARPC2	Actin-related protein 2/3 complex subunit 2	Arpc2	↑	↑	↓
ODO1	2-Oxoglutarate Dehydrogenase	Ogdh	↑	↑	↓
ARC1A	Actin-related protein 2/3 complex subunit 1A	Arpc1a	↑	↑	↓

### GO functional annotations and KEGG pathway analyses

**Figure 4:**
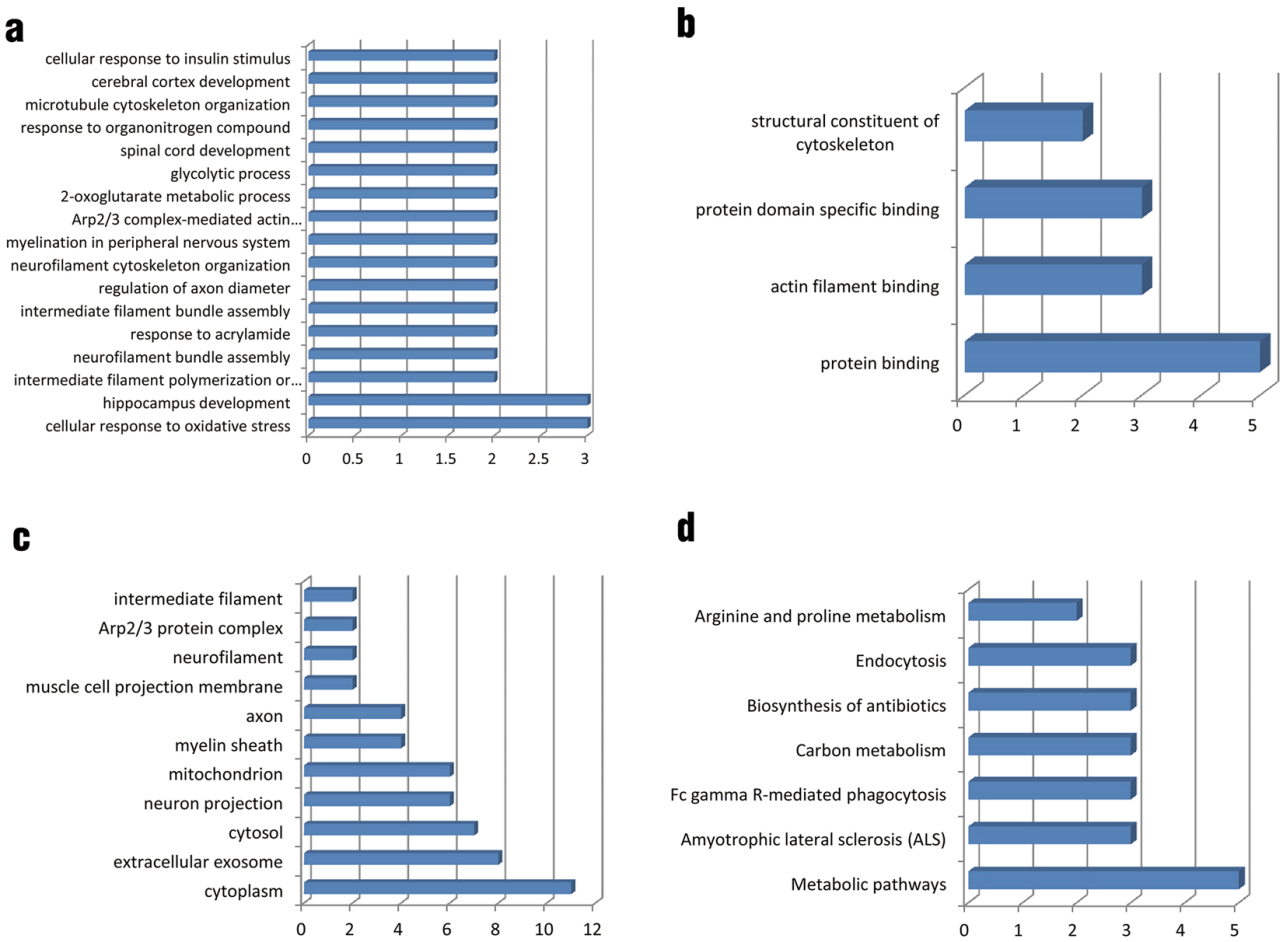
Effects of nimesulide on hippocampus proteomics in CUMS rats. (**a–c**) Functional annotations generated by GO analysis: biological process (**a**), molecular function (**b**) and cellular component (**c**). (**d**) Significant pathways generated based on the KEGG database.

GO functional annotations and KEGG pathway analyses were used to examine the differential proteins. The results show that, in terms of biological process, differential proteins were mainly involved in the cellular response to oxidative stress (8.33%) (Fig. 4a). Regarding molecular function, differential proteins were annotated as being associated with protein binding (38.46%), actin filament binding (23.08%), protein domain specific binding (23.08%), and structural constituent of the cytoskeleton (15.38%) (Fig. 4b). For cellular component, differential proteins are predicted to be in the cytoplasm (20.37%), extracellular exosome (14.81%), cytosol (12.96%), neuron projection (11.11%) and mitochondrion (11.11%) (Fig. 4c). Based on the KEGG database, the differentially expressed proteins were enriched in the following pathways: metabolic pathways, amyotrophic lateral sclerosis (ALS), Fc gamma R-mediated phagocytosis, carbon metabolism, biosynthesis of antibiotics, endocytosis and arginine and proline metabolism (Fig. 4d).

## Discussion

Rodent animal models for CUMS has been widely used for investigating the pathophysiological mechanism of depression and evaluating the efficacy of antidepressants^27^. This stress-induced model of depression has good validity and reliability^28^. Recent research has identified that depression has been linked with inflammatory markers and be associated with regulating genes of the immune response^29–31^. COX-2 is a key mediator of the inflammation, being responsible for the production of PGE2 from arachidonic acid and mediates different physiological and pathophysiological reactions via its receptors^32^. In the present study, we observed the depressive-like behavior emerges and an increased level of the proinflammatory mediator in a rat model of CUMS-induced depression. In the hippocampus, we screened differentially expressed proteins between rats with and without CUMS exposure.

Nimesulide, already introduced as an anti-inflammatory agent, was recognized to be a COX-2 selective inhibitor^33^. Recently, the effects of COX-2 inhibitor have been examined in various clinical trials, suggesting that it reduces cerebral damage and neuronal death^34,35^. Reported data from clinical trials supported the protective effects of the COX-2 inhibitor in depression^36^. Similar findings have been documented in animal models of chronic stress^37^. Our results agreed with previous reports that nimesulide could relieve depressive-like behaviors and reverse the increase of inflammatory factors. The more interesting result was that the overlappings differentially expressed proteins regulated by nimesulide were screened in CUMS rats.

To clarify the functions and links among these proteins, GO annotation enrichment analysis and KEGG signaling pathway enrichment analyses were used. GO annotation shows that differential proteins were involved in the formation of cellular components, such as cytoplasm and extracellular exosome. For molecular function, they exert binding function and comprise the cytoskeleton. In biological processes, it is mainly involved in regulating the cellular response to oxidative stress and hippocampus and cortex development. KEGG pathway analyses suggest that differentially expressed proteins are mainly involved in metabolic and endocytosis pathways. As mentioned above, the pathogenesis of depression involves several systems and multiple proteins. Our findings showed that these differentially expressed proteins could regulate by nimesulide and may be potential drug targets for depression treatment. Remarkably, we found these proteins might be related to inflammation, such as Adaptor protein two complex subunit beta 1 (Ap2b1), Calcineurin B type 1 (Cnb1), 2-Oxoglutarate Dehydrogenase (Ogdh), Calretinin (Calb2) and NAD-dependent protein deacetylase sirtuin-2 (Sirt2).

Among the changes evoked by nimesulide in the hippocampus, we observed an increase in the level of Ap2b1. Ap2b1, a subunit of the AP-2 complex, is involved in clathrin-mediated endocytosis and migration^38,39^. The knockdown of AP2b1 lessened the number of dendrites in developing rat hippocampal neurons^40^. Recently research found that Ap2b1 might exert neuroprotection through inhibiting inflammatory reaction^41^.

Of the upregulated proteins after nimesulide treatment, the expression of Cnb1 was notable. Cnb1, a regulatory subunit of calcineurin, equally distributed between the cytoplasm and nucleus^42^. Studies reported that Cnb1 showed increased expression after venlafaxine treatment^43^. Notably, calcineurin interacts with the serotonin transporter modulating its plasma membrane expression and serotonin uptake^44^. Moreover, calcineurin has also direct antidepressant-like effects, while inhibition of calcineurin in the medial prefrontal cortex of rats induces depressive-like behaviour^45^. Variants in Cnb1 are associated with a more rapid functional decline in Alzheimer’s disease^46^. Calcineurin-inhibitor agents also affect the activation of T cells, inhibit the expression of interleukins, and prevent the release of cytokines and inflammatory mediators from mast cells^47^.

In the present study, we found that nimesulide increased Ogdh level, known as a key enzyme in the tricarboxylic acid (TCA) cycles. Ogdh can catalyze the oxidative decarboxylation of α- ketoglutarate to form succinyl coenzyme A and release energy and it is known that energy deficiency would lead to dysfunction of organs and tissues, and then Alzheimer’s disease, Parkinson’s disease, and other diseases^48^. Besides, Ogdh is also closely related to chronic inflammation^49^. In short, Ogdh is speculated to be closely related to neuropsychiatric diseases and inflammation.

We noted the decrease in the expression of Calb2 after nimesulide administration. Calb2 is an important calcium-binding protein, which is mainly distributed in central neurons in the cerebral cortex and hippocampus^50^. Calb2 prevented calcium overload by binding excessive Ca^2+^, and thus reduce cell apoptosis^51^. Proteomics analysis in a global ischemic stroke also found that Calb2 associated with endoplasmic reticulum stress-induced neuronal cell apoptosis^52^.

Treatment for nimesulide decreased the level of Sirt2 in our study. Sirt2 is an NAD^+^-dependent deacetylase in the brain. The pharmacologic inhibition of Sirt2 exerts neuroprotective effects in diverse models of neurodegenerative disease^53,54^. Studies have shown that microtubule acetylation is essential for normal neuronal development and function^55,56^ and Sirt2 may function as the predominant microtubule deacetylase in mature neurons^57^. Sirt2 was found to be upregulated in response to oxidative stress, deacetylated FOXO3, and increased in the expression of genes targeted by FOXO3a, such as Bim^58^. Although Sirt2 is reported to a major inhibitor of microglia-mediated inflammation and neurotoxicity^59^, we cannot rule out the possibility that sirt2 also expression regulated by nimesulide through oxidative stress in the present research.

Summing up, our results suggest that nimesulide could improve depressive-like behaviors, suppress high levels of inflammatory factors, and regulate differentially expressed proteins in a rat model of CUMS-induced depression. Even though our findings highlight the role of nimesulide in depression and provide a promising therapeutic direction, the exact therapeutic mechanism behind the antidepressant effects of nimesulide has not been elucidated and future study is needed to explore the mechanism of action.
